# Case report: COVID-19-associated mucormycosis co-infection with *Lomentospora prolificans*: The first case and review on multiple fungal co-infections during COVID-19 pandemic

**DOI:** 10.3389/fmed.2023.1078970

**Published:** 2023-03-16

**Authors:** Mahzad Erami, Hossein Mirhendi, Mansooreh Momen-Heravi, Alireza Sharif, Seyed Jamal Hashemi Hezaveh, Amir Hassan Matini, Amir Hossein Ahsaniarani, Shima Aboutalebian

**Affiliations:** ^1^Department of Medical Parasitology and Mycology, School of Public Health, Tehran University of Medical Sciences, Tehran, Iran; ^2^Department of Infectious Diseases, School of Medicine, Infectious Diseases Research Center, Kashan University of Medical Sciences, Kashan, Iran; ^3^Department of Medical Parasitology and Mycology, School of Medicine, Isfahan University of Medical Sciences, Isfahan, Iran; ^4^Mycology Reference Laboratory, Research Core Facilities Laboratory, Isfahan University of Medical Sciences, Isfahan, Iran; ^5^Department of Pathology and Histology, School of Medicine, Shahid Beheshti Hospital, Kashan University of Medical Sciences, Kashan, Iran; ^6^Department of Otorhinolaryngology, School of Medicine, Matini Hospital, Kashan University of Medical Sciences, Kashan, Iran

**Keywords:** *Lomentospora prolificans*, COVID-19, mucormycosis, multiple co-infection, antifungal susceptibility testing

## Abstract

Along with the pandemic COVID-19 spreads, new clinical challenges have emerged in the health care settings, among which there is a high risk of secondary invasive fungal infections with significant mortality. Here, we report a case of invasive fungal rhino orbital sinusitis due to the simultaneous co-infection by *Rhizopus oryzae* and *Lomentospora prolificans*, both identified by sequencing, in a 70-year-old Afghanistanian female with COVID-19. The patient was subjected to surgical debridement as well as taking liposomal amphotericin B, voriconazole, and on discharge, her condition was good. As far as we know, this is the first case of co-infection of COVID-19-associated mucormycosis (CAM) and *Lomentospora prolificans* infection. Multiple fungal co-infections in COVID-19 patients are reviewed.

## Introduction

Most COVID-19 patients present with mild or moderate disease, however, patients with severe COVID-19, with comorbidities, or those receiving corticosteroid therapy and/or mechanical ventilation and intensive care, are predisposed to secondary opportunistic fungal infections (1) with high morbidity and mortality rate. Classical risk factors for invasive fungal infections in healthcare settings include prolonged neutropenia, hematologic malignancy, bone marrow, and solid organ transplantation, corticosteroid use, long stay in intensive care units (ICU), and uncontrolled diabetes mellitus (2).

Mucormycosis is an uncommon opportunistic life-threatening aggressive infection in humans and animals that is characterized by extensive angioinvasion that leads to vessel thrombosis and tissue necrosis (3), preventing the penetration of immune cells and antifungal agents to the infection site (4). The infection occurs in immunocompromised patients especially those with uncontrolled diabetes mellitus, neutropenia, immunosuppression, hematological malignancy, and hematopoietic stem cell or solid-organ transplantation (5). *Rhizopus* is the most common fungal agent of human mucormycosis in most case series, followed by *Mucor* and *Lichtheimia*, accounting for 70 to 80% of all mucormycosis cases (6, 7). Infections due to other genera such as *Rhizomucor*, *Apophysomyces*, *Saksenaea*, *Cunninghamella*, *Cokeromyces*, and *Syncephalastrum* are very rare (5).

*Lomentospora prolificans* (formerly *Scedosporium prolificans*) is an emerging invasive fungal opportunist that affects immunocompromised patients and even immunocompetent individuals (8), with a predilection for skin, sinuses, lungs, and central nervous system (9). The respiratory tract is considered the main route of entry for lomentosporiosis, but it may also occur *via* contaminated catheters (10). The infection is difficult to treat and has a high mortality.

There are numerous reports on co-infections of COVID-19 with fungal pathogens, but the complication of two filamentous fungal co-infections are rarely reported. As awareness and early diagnosis of such co-infections are important to initiate appropriate antifungal therapy and to prevent death (11), here, we report a simultaneous multiple co-infection by the filamentous fungi *Rhizopus oryzae* and *Lomentospora prolificans* in a COVID-19 patient. To better understand the clinical characteristics of such infrequent symbiosis between human fungal pathogens, we have reviewed the reported lomentosporiosis and the multiple fungal co-infections in COVID-19 patients.

## Case report

**Figure 1:**
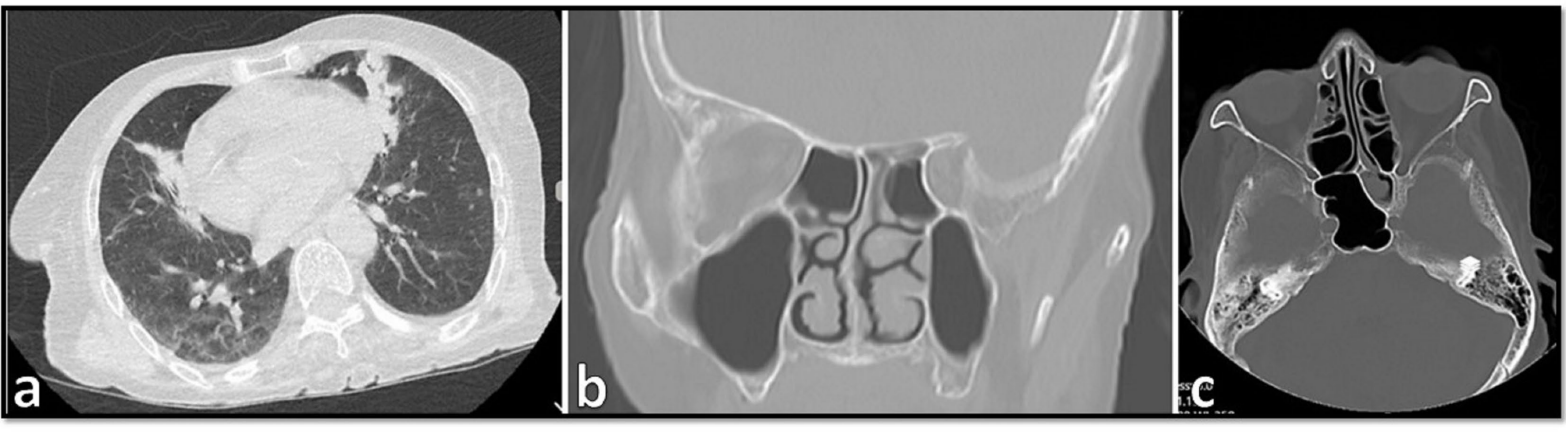
**(A)** Computed tomography scan of the chest in the case showing bilateral ground-glass opacities with a predominantly peripheral and multilobar location. **(B,C)** Orbital computed tomography scans of paranasal sinuses demonstrating opacification and air-fluid level in the left sphenoid sinus and some anterior ethmoid sinus air cells in right septal deviation to the right mucosal thickening in the left maxillary sinus.

On 29 November 2021 (day 1), a 70-year-old Afghanistanian female suspected of COVID-19 was hospitalized at Shahid-Beheshti Hospital, Kashan, Iran. She had a one-month history of headache, dizziness, dry cough, dyspnea, hemoptysis, myalgia, and severe weakness. In the physical examination, swelling over the right side of her face and numbness of the right side of her upper lip, a body temperature of 36°C, blood pressure of 90/60 mm Hg, respiratory rate of 18 breaths per minute, and oxygen saturation of 90% while the patient was breathing ambient air, were observed. Laboratory investigations were as followed: fasting blood sugar 76 mg/ dL (70–115 mg/dL), hemoglobin A1C 5.6% (Non-diabetic: 4.4–6.7%), sodium 135 mmol/L (135–145 mmol/L), potassium 4.1 mmol/L (3.5–5.3 mmol/L), calcium 8.6 mg/dL (8.6–10.6 mg/dL), blood urea nitrogen 29 mg/dL (7–23 mg/dL), creatinine 1.4 mg/dL (0.4–1.5 mg/dL), erythrocyte sedimentation rate (ESR) 84 mm in the 1st h, hemoglobin 11.6 g/dL (11.7–15.5 g/dL), white cell count 11.7 cells/μL (4–11 cells/μL), lymphocyte count 8.1 cells/μL (18–44 cells/μL), and C-reactive protein (CRP) 130 mg/L (normal <8). Her laboratory blood analysis revealed lymphopenia and a significant increase in CRP. Blood and urine cultures for fungi and bacteria were negative. Nasopharyngeal and oropharyngeal swabs were obtained and subjected to reverse transcription real-time polymerase chain reaction (rt-RT-PCR) for SARS-CoV-2 targeting the N and RdRp genes (Pishtaz Teb, Tehran, Iran) tested by Light Cycler 96 system (Roche, Germany) (12). The sample was positive with a high viral load (Ct value = 14.84 and 13.30 for the two targets). In computed tomography (CT) scan of the chest, bilateral ground-glass opacities with a predominantly peripheral and multilobar location were seen. The largest infiltrate was found in the base of the left lung with a tendency to consolidate, and in the right lung, opacities were located mainly in the upper lobe. Bilateral pleural thickenings were also not reported (Figure 1A).

Since her admission, the patient received atorvastatin (40 mg), levomepromazine (7.5 mL), and valacyclovir (500 mg). After the CT scan, and after the positive PCR results, hydroxychloroquine (400 mg/12 h), *remdesivir* (100 mg, 6 dosages), azithromycin (500 mg/day), dexamethasone (8 mg intravenously/twice daily) and vancomycin (IV, 1 gr) were added. On day 2, the patient was transferred to the ICU due to the exacerbation of hypoxia and respiratory distress and received mechanical ventilation assistance with orotracheal intubation. Due to the progress of hypoxia and the increase of inflammatory biomarkers, and the onset of the cytokine storm, dexamethasone was stopped and methylprednisolone (125 mg/day) was started for 3 days. After 3 days, the patient developed right periorbital edema and necrosis of the hard palate, and due to the recent increased prevalence of CAM among COVID-19 patients in the hospital, intravenous liposomal amphotericin B (5 mg/kg/day) was started as empirical treatment. CT scans of paranasal sinuses and orbital demonstrated opacification and air-fluid level in the left sphenoid sinus, and some anterior ethmoid sinus air cells in the right septal deviation to the right mucosal thickening in the left maxillary sinus. According to the mucosal thickening and accumulation of secretions in paranasal sinuses (Figures 1B,C), the patient underwent endoscopic sinus and nasal surgery on day 7. Endoscopy revealed black necrotic lesions and tumefaction localized in the right middle meatus. Necrotic lesions were mostly found in the maxillary and the ethmoidal sinuses. Necrotic tissue and a part of its surrounding healthy tissue were removed by surgery.

Although there was not enough evidence that the patient was immunocompromised, enough immunological tests to understand her defense status were not performed, therefore, the immunological status of the patient is unclear.

**Figure 2:**
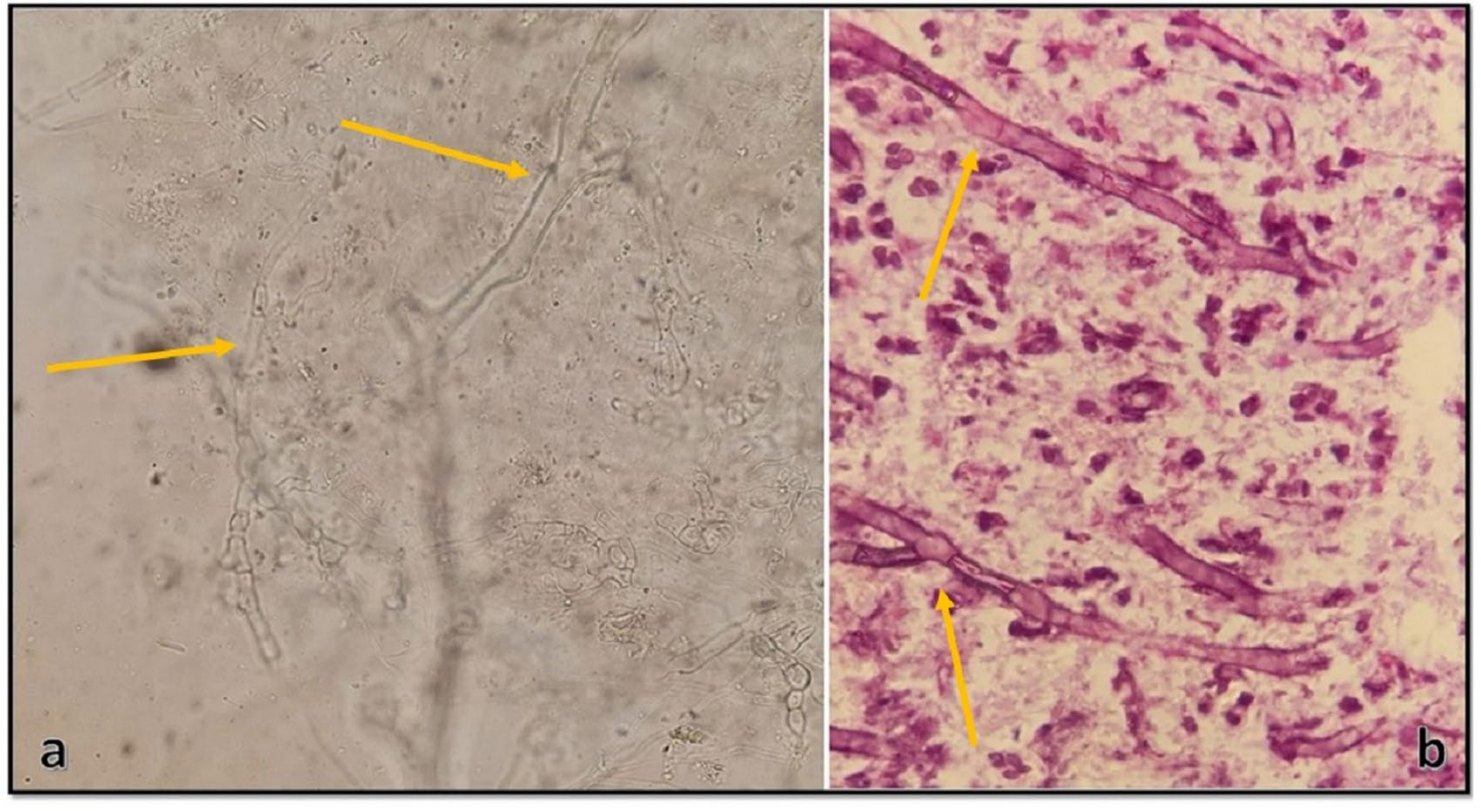
**(A)** Direct KOH microscopy of tissue samples. ‌Broad aseptate hyphae with wide-angle branches, and narrow septate hyaline hyphae with acute branching, are seen (×400). **(B)** Histopathological examination of tissue sections revealing a vascular structure involved, and containing both broad and thin fungal hyphae (×1,000).

Samples from sinusitis and tissue necrosis were taken and subjected to direct microscopy using KOH preparation and also to H&E histopathological examination, and broad aseptate and narrower septate hyaline hyphae were seen in both of them (Figures 2A,B), suggestive of proven invasive mucormycosis and hyalohyphomycosis. Therefore, voriconazole 400 mg/12 h on the first day and then 200 mg/12 h were added to the treatment. On day 12, PCR for SARS-CoV-2 was negative, and the same result was obtained again on day 20. Since then, the patient’s respiratory status improved markedly; she had no fever and the leukocyte count was lower than 11 cells/μL. On day 20 and day 23 the patient was discharged from ICU and the hospital, respectively. The patient began a 6-week course of oral voriconazole with close clinical and radiological surveillance. She was followed up for 15 weeks, during which her presenting symptoms have resolved, and is doing well.

**Figure 3:**
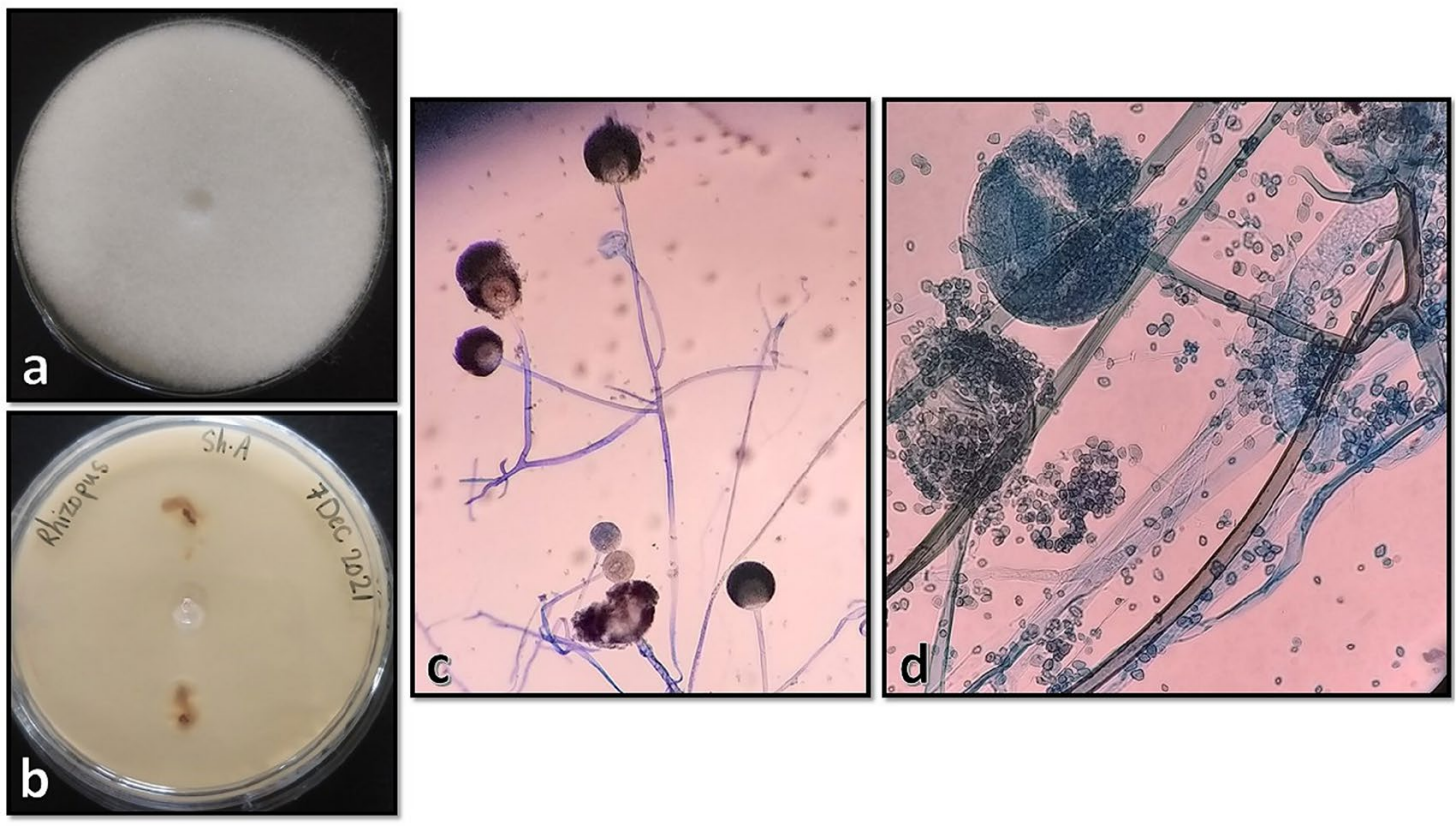
Surface and reverse of the isolated *Rhizopus oryzae* colony on Sabouraud dextrose agar after 4 days of incubation at 25°C **(A,B)**, and its microscopic morphology by ×100 and ×400 magnifications, respectively **(C,D)**.

**Figure 4:**
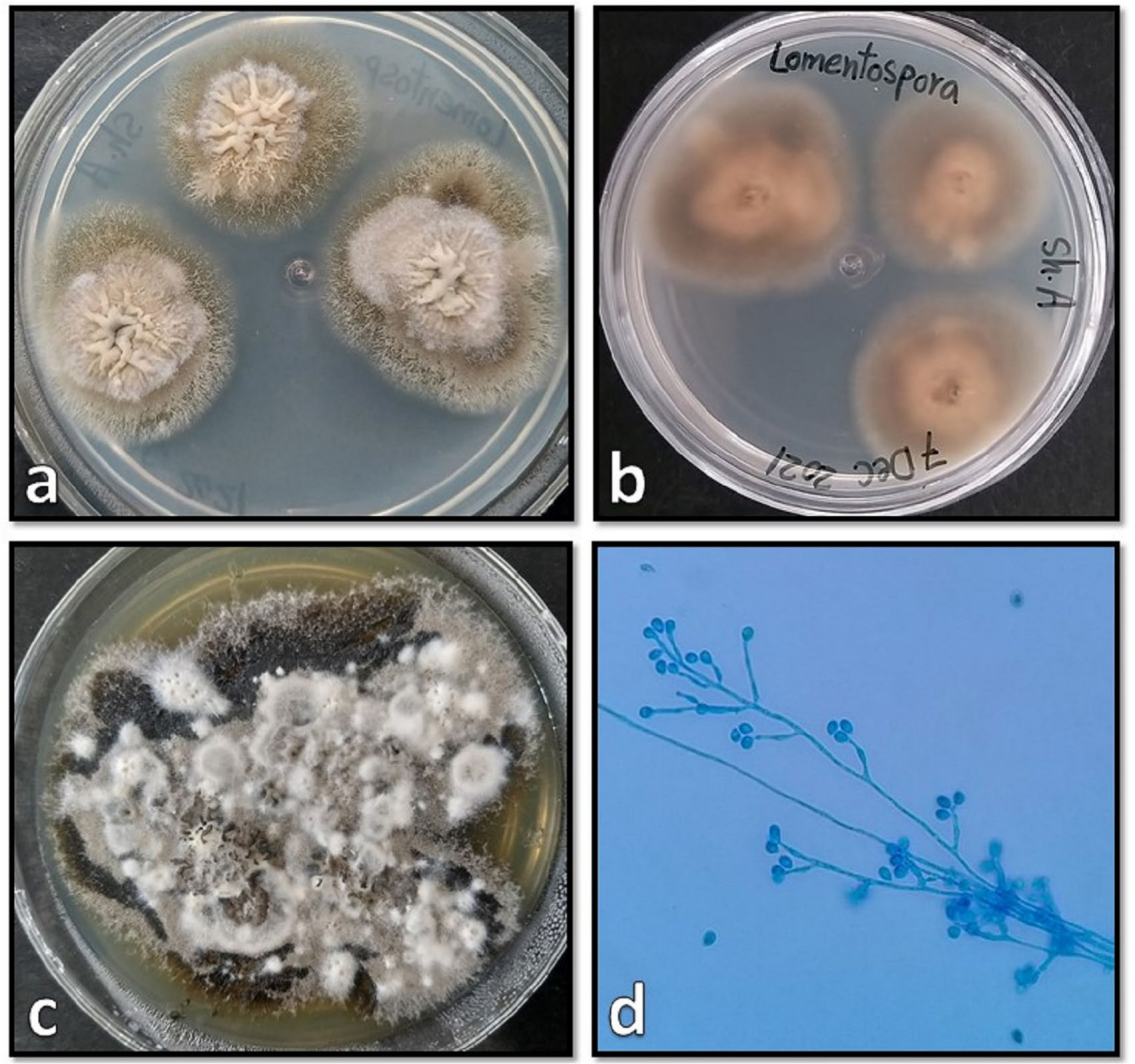
Surface and reverse of the isolated *Lomentospora prolificans* colony on Sabouraud dextrose agar after 4 days **(A,B)**, and after 20 days **(C)** incubation at 25°C when the mature colonies became dark gray to black and developed white mycelial tufts, and its microscopic morphology **(D)**.

Tissue samples were cultured on two Sabouraud dextrose agar (Merck, Germany) plates supplemented with 50 mg/L chloramphenicol with three inoculums on each plate, which resulted in the development of two different colonies on both plates. One of the colonies quickly covered the agar surface with dense growth, was cotton candy-like, white at first and then gray or yellowish-brown (Figure 3A), and its reverse was white to pale shades of gray or brown (Figure 3B). In the microscopic study of the slide culture prepared from the pure isolated colonies, broad hyphae with no or very few septa, numerous stolons running among the mycelia, and connecting groups of long sporangiophores were seen (Figures 3C,D). The other isolated colonies were cottony or moist and light gray to black (Figure 4A), and the reverse was gray to black (Figure 4B). The mature colony became dark gray to black and developed white mycelial tufts over time (Figure 4C). In slide culture preparation, septate hyphae, conidiogenous cells (annelids) having a swollen base and elongated neck, and small clusters of olive to brown, one-celled, smooth, and ovoid with a slightly narrowed, truncated base conidia at the apex were observed (Figure 4D). For molecular identification, DNAs of the colonies were extracted using physical destruction by glass-bead manipulation followed by phenol-chloroform purification as described previously (13), and the ITS1-5.8S-ITS2 region was PCR-amplified using the universal primers ITS1 and ITS4 (14). The PCR products were purified and Sanger-sequenced by the forward primer (Core Facilities Laboratory, Isfahan University of Medical Sciences, Iran). Based on NCBI and ISHAM barcoding databases, the isolates were identified as *Rhizopus oryzae* and *Lomentospora prolificans* with 100% sequence identity. The sequences were deposited in GenBank under the accession numbers ON220152 and ON220151, respectively.

Antifungal susceptibility testing (AFST) was performed for *Rhizopus oryzae* and *Lomentospora prolificans* isolated from the patient, based on the Clinical and Laboratory Standards Institute (CLSI, M38-A2) method for amphotericin B, isavuconazole, posaconazole, itraconazole, voriconazole, fluconazole, caspofungin and terbinafine. The minimum inhibitory concentrations (MIC)/ minimum effective concentration (MEC) were determined visually after 24 and 72 h of incubation, respectively. *Candida parapsilosis* ATCC 22019 was used as the quality control strain. The results of AFST are seen in Table 1.

**Table 1:** The results (μg/mL) of antifungal susceptibility testing of *Rhizopus oryzae* and *Lomentospora prolificans.*

	AmB	ISV	POS	ITC	VRC	FLC	CAS	TER
*Rhizopus oryzae*	0.5	2	0.25	2	16	>64	>8	>16
*Lomentospora prolificans*	>16	8	>16	>16	8	>64	>8	>16

AmB, Amphotericin B; ISV, Isavuconazole; POS, Posaconazole; ITC, itraconazole; VRC, Voriconazole; FLC, fluconazole; CAS, Caspofungin; TER, terbinafine.

## Discussion

This is the first case of COVID-associated mucormycosis (CAM) co-infection with *Lomentospora prolificans* during the global epidemics of SARS-CoV-2. The use of steroids in hospitalized COVID-19 patients and the associated risk of hyperglycemia lead to an increased risk for secondary infections such as invasive aspergillosis and mucormycosis (15). There is an increase in the development of invasive fungal infections in patients who received corticosteroid therapy compared to patients who did not receive steroids (16, 17). In our case, dexamethasone and methylprednisolone were incorporated into cure protocols for COVID-19 infection. The cumulative dose of glucocorticoids was 391 mg. Muthu et al. reported that the cumulative glucocorticoid dose is a contributory factor for CAM. Therefore, in hypoxemic COVID-19 patients, glucocorticoids should be used with caution (16), and should be avoided in patients without hypoxemia (18).

*Scedosporium* spp./*Lomentospora* spp., and *Fusarium* spp. are also emerging rare opportunistic pathogens in immunocompromised individuals with an incidence rate 3–8 times lower than the Mucorales (19). The prognosis of the infections caused by *L. prolificans* is poor. *L. prolificans* can be recognized by direct microscopy and culture, however, laboratories are not familiar with its morphology. In addition, *in vitro* antifungal susceptibility studies confirm that *L. prolificans* is intrinsically resistant to current available systemic antifungal agents (20) and the disease is often refractory to treatment, yielding high mortality rates of up to 90% (21). It is reported that a combination of terbinafine with miconazole, voriconazole, or itraconazole has synergy against *L. prolificans* (22). Also, combinations of voriconazole plus either liposomal amphotericin B or micafungin are moderately supported. The ESCMID guidelines (2014) recommended a combination therapy containing voriconazole and terbinafine for *L. prolificans* infections (23). In a study, five out of eight patients with *Lomentospora* infections, who received the combination therapy with voriconazole plus at least one other agent (in 4/5 patients the combination included voriconazole and terbinafine), but no patients who received monotherapy, survived (24). In our case, liposomal amphotericin B, voriconazole and surgical debridement were used, and in surgery not only necrotic tissue but also healthy surrounding tissue were removed to prevent spreading to other tissues.

The epidemiology and outcomes of non-*Aspergillus* invasive mold infections in 3 years between 2014 to 2017 at a university hospital in San Diego, California, United States, were reviewed (24), in which eight cases of *Lomentospora prolificans* were found, two of them were mixed with *Scedosporium apiospermum* and *Mucor* sp. (24). Also, Lamaris et al. (25) reported a case of co-infection with *Scedosporium* and *Rhizopus*, but no clinical information is available. The global epidemiological status of lomentosporiosis reported between 2000 to 2018 from more than 74 different countries was reviewed (21), which 56 patients with *L. prolificans* infection were identified in Australia (*n* = 14), Japan, the United States (each *n* = 8), Spain (*n* = 7), France, Germany (each *n* = 5), India, Italy, United Kingdom (each *n* = 2), Brazil, Netherlands, and Poland (each *n* = 1). Males (*n* = 32, 57.14%) were more frequently infected than females, ranging from 42 to 67 years. Three out of 56 were mixed with another mold including *Aspergillus* spp.*, Exserohilum* spp., and *Scedosporium apiospermum* (21). While *L. prolificans* is considered an emerging pathogen, it is unknown if it is restricted to some countries in Europe, Australia, and some Southern states of the USA (11) or if the diagnostic conditions have influenced the epidemiology. As far as we know this is the first case of *L. prolificans* isolated from human specimens in Iran.

There are numerous reports on co-infections of COVID-19 with fungal pathogens, but the complication of multiple filamentous fungal co-infections are rarely reported. As listed in Table 2, fungus-fungus co-infections were mostly observed as pulmonary (*n* = 14, 51.85%) followed by sinusitis and rhinosinusitis (each *n* = 3, 10.7%) in which the frequency of *Aspergillus*-Mucorales (*n* = 13, 46.42%) and *Aspergillus-Aspergillus* (*n* = 12, 42.85%) coexistence was almost similar. The present case is the first report of multiple co-infections of *R. oryzae* and *L. prolificans* in COVID-19 patients. We reviewed 27 cases (including the present case) of multiple fungal co-infections published during the COVID-19 pandemic. Only six of the 27 patients were healthy, while the rest were diabetic or had other underlying systemic illnesses. Males (*n* = 17, 62.96%) were more frequently infected than females and the age range was 31–82 years old, mostly 50–70 years (*n* = 20, 74.07%). Most patients were treated with amphotericin B, liposomal amphotericin B, or voriconazole; 17 of 27 survived and 10 died among which only two were previously healthy. The clinical overview of patients with multiple fungal co-infections in COVID-19 is summarized in Table 2.

**Table 2:** Clinical overview of patients with multiple fungal co-infections in COVID-19.

Case no.	Study	Geography	Number of cases	Age/sex	Underlying diseases	Type of infection	Antifungal	Co-infection	Outcome	Ref
1	Nasir N, 2020	Pakistan	2	57/M	DM/HTN	Pulmonary	AmB	*A. flavus + A. fumigatus*	Died	(26)
				55/M	DM/Gas gangrene	Septic shock, MODS	No antifungal	*A. flavus* + *A. niger*	Died	
2	Zayet S, 2020	Tunisia	1	69/F	HTN, DM	Rhino-orbito- Cerebral	AmB deoxycholate, VRC	*A. flavus + Rhizopus spp.*	Cured	(27)
3	Machado M, 2020	Spain	1	63/M	HTA, CKD, asthma	Pulmonary	Isavuconazole	*A. fumigatus + A. awamori + A. terreus*	Died	(28)
4	Marr KA, 2021	Spain	2	70/M	HTN	Pulmonary	VRC, POS, L-AmB	*A. fumigatus + A. niger*	Cured	(29)
				76/M	COPD	Pulmonary		*A. fumigatus + A. niger*		
5	Moorthy A, 2021	India	1	45/M	Immunocompetent	Rhino-orbito- Cerebral	L-AmB, VRC, POS	*Aspergillus spp. + Mucorales*	Cured	(30)
6	Bellanger A-P, 2021	France	1	55/M	Auto-HCT	Pulmonary	L-AmB	*A. fumigatus* + *R. microsporus*	Died	(31)
7	Buil JB, 2021	Netherlands	2	late 50s/M	Immunocompetent	Pulmonary	VRC, L-AmB, POS	*A. fumigatus + Lichtheimia ramosa*	Died	(32)
				late 60s/M	CLL, DM, obesity	Pulmonary	VRC, L-AmB, ISV	*A. fumigatus* + *R. microsporus*	Died	
8	Anita A, 2021	India	3	53/M	Diabetes, HTN	Sinusitis and orbital	L-AmB, POS	*A. flavus + Rhizopus spp.*	Cured	(33)
				52/F	Diabetes	Sinusitis		*A. flavus + Rhizopus spp.*		
				63/M	Immunocompetent	Sinusitis		*A. flavus + Rhizopus spp.*		
9	Abolghasemi S, 2021	Iran	1	66/F	Immunocompetent	Pulmonary	VRC, CAS	*A. terreus + A. fumigatus*	Died	(34)
10	Martins AC, 2021	Brazil	1	68/F	DM / CKD / HTN	Pulmonary	VRC	*A. flavus + A. fumigatus*	Died	(35)
11	Johnson AK, 2021	USA	1	79/M	HTN, DM	Pulmonary	VRC, L-AmB	*A. fumigatus* + *R. oryzae*	Cured	(36)
12	Paramythiotou E, 2021	Greece	2	82/F	HTN, dementia	Pulmonary	ISV	*A. fumigatus + A. flavus*	Died	(37)
				66/M	Smoking, obesity, sleep apnea	Pulmonary		*A. fumigatus + A. terreus*	Cured	
13	White PL, 2021	Wales	1	NA	DM, HTN, obesity	Sinusitis	VRC	*A. fumigatus + A. versicolor*	Cured	(38)
14	Costache C, 2021	Romania	1	53/F	Diabetes	Pulmonary	VRC	*Aspergillus section Flavi* + *Aspergillus section Fumigati*	Cured	(39)
15	Tabarsi P, 2022	Iran	1	50/F	Diabetes	Rhinosinusitis	L-AmB	*A. flavus* + *A. niger*	Cured	(40)
16	Benhadid-Brahmi Y, 2022	France	1	74/M	HTN	Pulmonary	VRC, L-AmB	*A. welwischiae* + *R. delemar*	Died	(41)
17	Jawanda MK, 2022	India	1	70/M	Diabetes	Maxillary Osteomyelitis	POS	*Mucorales., Actinomycetes spp.*, *Candida* spp.	Cured	(42)
18	Tabarsi P, 2022	Iran	2	31/F	Immunocompetent	Rhinosinusitis	L-AmB, POS	*A. flavus* + *R. oryzae*	Cured	(43)
				58/M	Diabetes, HTN	Rhinosinusitis		*A. fumigatus* + *R. oryzae*	Cured	
19	Suresh A, 2022	India	1	57/M	Diabetes	Osteomyelitis of Jaws and Sinuses	L-AmB	*A. fumigatus* + *Mucorales*	Cured	(44)
20	Erami M, 2022	Iran	1	70/F	Immunocompetent	Rhino-orbital	L-AmB, VRC	*Lomentospora prolificans* + *R. oryzae*	Cured	This study

M, male; F, female; NA, not available; CKD, Chronic Kidney Disease; HTN, hypertension; COPD, chronic obstructive pulmonary disease; DM, diabetes mellitus, CLL, chronic lymphocytic leukemia; auto-HCT, autologous hematopoietic stem cell transplantation; MODS, Multi-organ dysfunction syndrome; L-AmB, Liposomal amphotericin B; AmB, Amphotericin B; VRC, Voriconazole; CAS, Caspofungin; POS, Posaconazole; ISV, Isavuconazole; NYS, Nystatin.

## Conclusion

We reported the first case of co-infection of COVID-19-associated mucormycosis with *Lomentospora prolificans* infection and reviewed the multiple fungal co-infections in COVID-19 patients, and also the cases of lomentosporiosis. Despite frequent prescriptions of broad-spectrum empirical antimicrobials in patients with COVID-19, there is a paucity of data to support the association with two filamentous fungal co-infections. Understanding the predictors of co-infection and multiple infections may help to determine the appropriate interventions to reduce mortality and morbidity. In addition, the generation of prospective evidence to support the detection, identification, development of antimicrobial policy, and appropriate stewardship interventions specific to the COVID-19 pandemic is required.

## Data availability statement

The datasets presented in this study can be found in online repositories. The names of the repository/repositories and accession number(s) can be found in the article/supplementary material.

## Ethics statement

The studies involving human participants were reviewed and approved by ethical approval of the study was obtained from the Ethics Committee of Tehran University of Medical Sciences, Tehran, Iran (IR.TUMS.SPH.REC.1399.329). The patients/participants provided their written informed consent to participate in this study. Written informed consent was obtained from the individual(s) for the publication of any potentially identifiable images or data included in this article.

## Author contributions

ME and SA performed all the experiments and participated in data collection. SA drafted the manuscript, participated in database searching and data extraction, and analyzed and interpreted the data. MM-H, SH, AS, AM, and AA participated in collecting the clinical isolate and data collection. HM supervised all parts of the study and critical review of the manuscript. All authors contributed to the article and approved the submitted version.

## Funding

This work was supported by Isfahan University of Medical Sciences, Isfahan, Iran (grant number 1400180).

## Conflict of interest

The authors declare that the research was conducted in the absence of any commercial or financial relationships that could be construed as a potential conflict of interest.

## Publisher’s note

All claims expressed in this article are solely those of the authors and do not necessarily represent those of their affiliated organizations, or those of the publisher, the editors and the reviewers. Any product that may be evaluated in this article, or claim that may be made by its manufacturer, is not guaranteed or endorsed by the publisher.
